# Gender differences on effectiveness of a school-based physical activity intervention for reducing cardiometabolic risk: a cluster randomized trial

**DOI:** 10.1186/s12966-014-0154-4

**Published:** 2014-12-10

**Authors:** Vicente Martínez-Vizcaíno, Mairena Sánchez-López, Blanca Notario-Pacheco, Fernando Salcedo-Aguilar, Montserrat Solera-Martínez, Pablo Franquelo-Morales, Sara López-Martínez, Jorge C García-Prieto, Natalia Arias-Palencia, Coral Torrijos-Niño, Ricardo Mora-Rodríguez, Fernando Rodríguez-Artalejo

**Affiliations:** Social and Health Care Research Center, Universidad de Castilla-La Mancha, Cuenca, Spain; Facultad de Ciencias de la Salud, Universidad Autónoma de Chile, ᅟ, Chile; School of Education, Universidad de Castilla-La Mancha, Ciudad Real, Spain; Cuenca I Primary Care Center, Cuenca, Spain; Hospital Virgen de la Luz, Cuenca, Spain; Exercise Physiology Laboratory, Universidad de Castilla-La Mancha, Toledo, Spain; Department of Preventive Medicine and Public Health, School of Medicine, Universidad Autónoma de Madrid, Madrid, Spain

**Keywords:** Physical activity, Intervention, Obesity, Cardiometabolic risk factors, Metabolic syndrome, Children

## Abstract

**Background:**

Studies that have examined the impact of a physical activity intervention on cardiometabolic risk factors have yielded conflicting results. The objective of this study was to assess the impact of a standardized physical activity program on adiposity and cardiometabolic risk factors in schoolchildren.

**Methods:**

Cluster randomized trial study of 712 schoolchildren, 8–10 years, from 20 public schools in the Province of Cuenca, Spain. The intervention (MOVI-2) consisted of play-based and non-competitive activities. MOVI-2 was conducted during two 90-minute sessions on weekdays and one 150-minute session on Saturday mornings every week between September 2010 and May 2011. We measured changes in adiposity (overweight/obesity prevalence, body mass index [BMI], triceps skinfold thickness [TST], body fat %, fat-free mass, waist circumference) and other cardiometabolic risk factors (LDL-cholesterol, triglycerides/HDL-cholesterol ratio, insulin, C-reactive protein and blood pressure). The analyses used mixed regression models to adjust for baseline covariates under cluster randomization.

**Results:**

Among girls, we found a reduction of adiposity in intervention versus control schools, with a decrease in TST (−1.1 mm; 95% confidence interval [CI] -2.3 to −0.7), body fat % (−0.9%; 95% CI −1.3 to −0.4), waist circumference (−2.7 cm; 95% CI −4.5 to −0.9), and an increase in fat-free mass (0.3 kg; 95% CI 0.01 to 0.6). The intervention also led to lower serum LDL-cholesterol and insulin levels. Among boys, a reduction in waist circumference (−1.4 cm; 95% CI −2.6 to −0.1; P = 0.03), and an increase in fat-free mass (0.5 kg; 95% CI 0.2 to 0.9; P = 0.003) was associated with the intervention versus control schools. The prevalence of overweight/obesity or underweight, BMI, and other cardiometabolic risk factors was not modified by the intervention. No important adverse events were registered.

**Conclusions:**

An extracurricular intervention of non-competitive physical activity during an academic year, targeting all schoolchildren regardless of body weight, is a safe and effective measure to reduce adiposity in both genders and to improve cardiometabolic risk profile in girls.

**Trial registration:**

Clinical trials NCT01277224.

**Electronic supplementary material:**

The online version of this article (doi:10.1186/s12966-014-0154-4) contains supplementary material, which is available to authorized users.

## Background

Obesity in children is an alarming public health problem in almost all the countries in the world [1]. In Cuenca province, Spain, children’s overweight/obesity prevalence is around 35% [2], an estimate similar to those of other geographical Spanish areas [3], Mediterranean countries [4] and the USA [5]. Obese children are at increased risk of becoming obese adults [6,7] and this tracking becomes stronger the closer the child gets to adult status [8]. Childhood obesity has been described as an independent predictor of coronary heart disease in adulthood [9] and Metabolic syndrome (MetS), although evidence for the latter association are not as consistent [10].

Approximately half of the reported interventions for preventing obesity in schoolchildren have shown a significant effect on some categorical or continuous measure of fatness [11]. Despite the close relationship between adiposity and MetS, the school-based physical activity (PA) interventions including MetS or cardiometabolic risk as an end-point are scarce and their results mixed. Moreover, while in all the interventions focused on aerobic exercise, significant improvements have been observed in at least one of the insulin-related variables examined, in only one out of the four interventions that were focused on muscular strength were reported significant improvements in any of these variables [12]. These controversial results may be attributed, at least in part, to three reasons related to the lack of statistical power: the very low prevalence of MetS in children, the modest sample size of the studies, and finally, the lack of a single sensitive measure of MetS. Moreover, it appears surprising this predominant approach to aerobic physical activity when it has been reported that associations between cardiorespiratory fitness and strength with insulin resistance and b-cell function were independent of each other in young people [13] and also in children [14,15]; therefore it seems evidence based supported that physical exercise interventions in these age groups should target separately aerobic activities and muscle strengthening activities.

Furthermore, interventions with a favourable impact on some cardiometabolic variables were school-based and usually entailed modifications of the physical education curriculum [16-19]. Unfortunately, changes in the school curriculum are not allowed in Spain and many other countries. In a previous study, we demonstrated that an extracurricular non-compulsory PA intervention reduced adiposity and improved blood lipids in schoolchildren [20]. However, as in other studies [17,21], the intervention may not have reached its maximum potential effectiveness given that higher levels of PA during the intervention could have been partially offset by more sedentary behaviours outside the intervention. Finally, a frequent limitation in the literature in this field is the paucity of information on the intervention process which hinders replication and sustainability of the intervention [11].

In the school environment, non-competitive recreational activities provide motivating learning experiences to discover the pleasure of moving and are appropriate for all schoolchildren regardless of their level of motor skill, gender or weight status. These characteristics of some school interventions have demonstrated effectiveness in reducing adiposity [20].

Our study assessed the impact of a standardized PA program on adiposity and cardiometabolic risk in fourth- and fifth-grade schoolchildren. The program consisted of non-competitive recreational activities focused on developing aerobic and muscular fitness. We also report extensive data on the intervention process.

## Methods

### Study design, setting, and participants

The study methods have been reported in detail elsewhere [22]. The study followed recommendations of the CONSORT statement on cluster randomized trials [23], and was registered in ClinicalTrials.gov (registration number NCT01277224). Briefly, a cluster-randomized trial was conducted to prevent contamination between intervention and control participants. This trial included 20 schools in 20 towns in the Province of Cuenca, Spain. All but two were rural schools (located in towns less than 5,000 inhabitants).

### Randomisation and blinding

Using a computer generated procedure; ten schools were randomized to the intervention group (IG), and ten to the control group (CG). In towns with two or more schools, only one was chosen at random to avoid contamination of the intervention. Briefly, we conducted a field trial in which 20 schools (clusters) were randomly allocated (by using opaque envelopes) to either the IG or the CG. In the intervention schools, the PA program (MOVI-2) was implemented during one academic year, while the control schools kept their usual patterns of PA. Schools were informed of the result of randomization after they agreed to participate in the study. Blinding of the school allocation was done for the laboratory determinations but not for other outcome variables, because they were measured in the school setting. Variables used to evaluate the effectiveness of MOVI-2 were measured in the two trial groups at the beginning (September 2010) and at the end (June 2011) of the intervention.

### Study participants

All the children in the fourth and fifth grades in the 20 selected schools were considered eligible for study inclusion if they met the following criteria: a) literacy in Spanish; b) lack of serious learning difficulties or of physical and mental disorders identified by parents and teachers, which could impede participation in the scheduled activities, and c) absence of any chronic disease that, as judged by their pediatrician or family doctor, would preclude participation in MOVI-2. The collaboration of a family member who would respond to questionnaires on lifestyle was also required.

### Ethics

The School Boards (community participatory committee in each school) and the children’s parents were informed of the study’s aims and methods, and were asked to confirm approval of the study in writing. The study was also presented classroom-by-classroom to the children, and their oral consent was obtained. The Clinical Research Ethics Committee of the Cuenca Regional Health Authority approved the study protocol, and an insurance policy was contracted to cover injuries during the PA program.

### Intervention

MOVI-2 was based on a socio-ecological model, which targeted the school, parents, teachers and children, and focused on increasing PA [24]. MOVI-2 consisted of an extracurricular play-based and non-competitive PA program. The primary objective of MOVI-2 was to increase weekly PA and to improve health-related fitness. MOVI-2 included basic sports games, traditional games, and other outdoor activities such as cycling or gymkhanas (http://www.movidavida.org/). The program included two 90-minute PA sessions during the weekdays in the evening from 4 to 5.30 pm and one 150-minute session on Saturday morning each week. In the weekday sessions there was a break of five minutes and in the Saturday session were there two breaks of five minutes where children could drink water. All activities were implemented by monitors with technical qualifications in PA and sports, physical education teachers, or PA science graduates, specifically engaged and adequately trained for the program.

The standard physical education curriculum (2 h per week of physical activity at low-to-moderate intensity) continued to be taught in both the control and intervention schools, because it is compulsory for all primary school pupils in Spain.

#### Compliance and monitoring

To improve adherence to MOVI-2, participants attending at least 70% of the sessions received small gifts depicting the logo of the program’s mascot as a reward. To stimulate positive attitudes toward PA, fair play and cooperation, coloured badges were also handed out.

Parents and teachers were informed about: a) the importance of cardiometabolic risk factors in childhood as predictors of cardiovascular disease and MetS in adulthood; b) the importance of PA for the children’s health; and c) the fact that the MOVI-2 program would be implemented by trained instructors. Specific information about strategies to promote parents’ involvement in MOVI-2 has been reported previously [22].

#### Study variables

##### Primary endpoints

The main endpoints (adiposity and cardiometabolic risk factors) and their measurement techniques have been reported elsewhere [22]. Weight was measured twice (Seca® 861 scales) with the child barefoot and in light clothing. Height was also measured twice, using a wall stadiometer (Seca® 222), with the child barefoot and upright and with the sagittal midline touching the back board. Waist circumference (WC) was measured 3 times at the midpoint between the last rib and the iliac crest at the end of a normal expiration and using a flexible tape. Triceps skinfold thickness (TST) was measured 3 times at the triceps using a Holtain Ltd. caliber (0.2 mm accuracy and consistent 10 g/mm^2^ pressure between valves). The body fat % and the fat-free mass were estimated with an eight-electrode BC-418 MA bioimpedance analysis system (Tanita Corp. Tokyo, Japan) [25]. Children were classified as normal weight, overweight or obese, according to body mass index (BMI) cut-off values proposed by Cole and Lobstein [26]. Systolic and diastolic blood pressures were measured with an OMRON-M5-I automatic tensiometer (Omron Healthcare UK Ltd.) [27]. Two readings were obtained after a 5-minute rest, with a 5-minute interval between measurements. The mean arterial pressure (MAP) was then calculated using the following formula: DBP + (0.333 × (SBP-DBP)). Anthropometry and blood pressure measurements were made by trained nurses.

Blood samples were taken in the morning between 8:15 and 9:00 h, after a 12-hour fast. The samples were processed using a Roche Diagnostics COBAS C711. The following parameters were determined: triglycerides (GPO-PAP enzymatic method), HDL-cholesterol and LDL-cholesterol (2nd generation method without deproteinisation), insulin (chemiluminescent microparticle immunoassay) and C-reactive protein (latex-enhanced nephelometry).

##### Process indicators

Energy expenditure attributable to each MOVI-2 game was estimated in 32 students from an intervention school, using oxygen consumption as measured by a portable gas analyser (Cosmed® K4b2, Rome, Italy) [28]. We monitored 40 sessions of the program conducted from February to May, from 4 to 5.30 pm. On each session, one of the participants was randomly selected and equipped with a portable indirect calorimetry system harnessed to their upper body. In addition, the child wore a pediatric mask covering his/her mouth and nose, a chest band transmitter to record heart rate (Polar; F1TM, Finland). Once equipped, the child was fully integrated into the exercise session and researchers did not interfere with the child activity. Out of the 40 sessions attended, two were discarded due to the lack of recorded values. Thirty-eight sessions were recorded in thirty-two different children, and six participants were tested in two sessions separated by at least one week.

All activities were performed indoors in the school’s gymnasium and require materials habitual in most European primary school gymnasium (soft rubber balls, road signal cones, flag waist bands, plastic gymnastic loops). Games were classified into two big categories: a) endurance games in which the main PA was running (i.e., chasing, sprinting, dribbling, hopping, and such) and b) resistance games in which there were also locomotion involving opposition from a partner (lifting, pushing, wrestling, hauling, and such). Each game session lasted approximately 90 min and included 9 games of 5.5 ± 1.4 min of duration interspersed by periods of 4 ± 1 min for recovery and organization.

Daily PA was evaluated using accelerometry in a subsample of 200 randomly selected children from eight of the participating schools (2 CG and 6 IG). An accelerometer (MTI/CSA 7164 device, ActiGraph®, Shalimar, Florida, United States) was used for seven consecutive days (and nights). Data were analysed using KineSoft software, version 3.3.2.0.

Intervention children and their parents also completed a questionnaire on satisfaction and compliance with the PA program. Finally, we evaluated the cost of MOVI-2 using standard procedures [29].

##### Potential confounders

Food consumption was estimated with a self-administered computerized 24-h dietary recall [30,31]. Because of the lower cognitive ability in children aged 8–9 years, the dietary recall was used only in fifth-grade children. The questionnaire was administered twice, on Monday (to evaluate weekend diet) and on one other school day (to evaluate working day diet); only subjects that completed the two recalls were included in the analysis. Finally, we obtained food consumption data of two non-consecutive 24-h recalls of 401 children, of which 320 completed all the measurements (79.8%).

Sexual maturity was obtained by a standardized procedure in which parents identified their children’s pubertal status using figures based on Tanner stages [32].

Parental employment status. Parents were asked about the highest parental employment status in the family (either mother or father) by means of a questionnaire.

### Statistical analysis

We analyzed the data using mixed regression models, where the dependent variable was each endpoint at the end of follow-up. Models were adjusted for the baseline values of each endpoint, age, Tanner change, and cluster (random effect). The intervention was included in the model as a fixed effect, using an independent dummy variable with a value of 1 for intervention schools, and 0 for control schools.

Results were expressed as the absolute difference in the change in endpoints from baseline to the end of follow-up between intervention and control schoolchildren, by sex. However, when the dependent variable was the prevalence of obesity or underweight, odds ratios (OR) with their 95% CI were estimated. We tested whether the effect of the intervention differed between boys and girls, by using interaction terms that were the product of the intervention by sex. Likewise, we tested if the impact of the intervention varied across categories of baseline BMI, Tanner stage and across categories of parental employment status, by using likelihood ratio tests, which compared models with and without interaction terms.

Analyses were performed according to intention-to-treat, with children analyzed in their original randomized allocation regardless of the number of MOVI-2 program sessions attended.

Statistical significance was set at P < 0.05. The analyses were performed with SAS, version 9.3 (SAS institute INC., Cary, North Carolina).

## Results

### Participation flow

**Figure 1:**
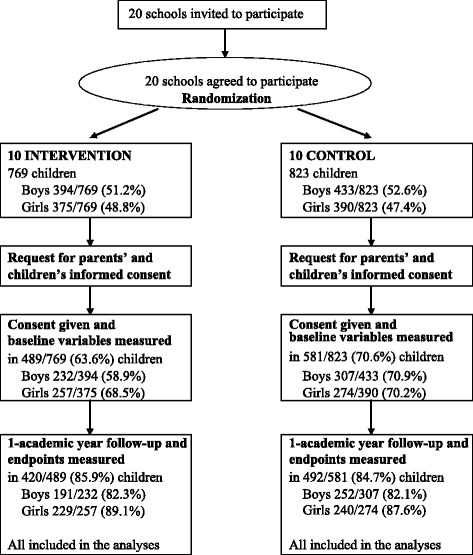
**Flowchart showing the progress of clusters (schools) and schoolchildren through the study.**

Figure 1 displays the flow of schools and participating children across the intervention study. All the schools invited agreed to participate. The age range of participating children was 8 to 11 years at study baseline. We found no differences by sex, age or adiposity measurements at baseline between the children who completed the study and those who did not. In both the intervention and control schools, participation rates at baseline and at the end of study were similar in boys and girls.

### Baseline data

The baseline characteristics of the study cohort are presented in Table 1. There were no statistically significant differences between intervention and control participants in any baseline characteristics.

**Table 1:** **Baseline characteristics of intervention and control schoolchildren, by sex**

	**Intervention group**		**Control group**	
	**Girls (n = 229)**	**Boys (n = 191)**	**Girls (n = 240)**	**Boys (n = 252)**
Age, mean ± SD, years	9.4 ± 0.7	9.4 ± 0.7	9.5 ± 0.7	9.5 ± 0.7
Born abroad, %	12.7	12.0	14.2	17.1
Birth weight, kg	3.2 ± 0.5	3.4 ± 0.5	3.2 ± 0.5	3.2 ± 0.4
Tanner stage, %				
Stage 1	61.2	55.9	66.0	62.5
Stage 2	30.4	42.8	26.7	33.3
Stage ≥3	8.4	1.3	7.3	4.2
Highest parental educational level, %				
Primary or lower	7.3	3.6	8.7	5.2
Secondary	65.1	81.0	73.3	75.2
University	27.5	15.5	18.0	19.6
Highest parental employment status, %				
Housewife, student or unemployed	9.0	7.1	9.2	9.2
Employee	38.7	54.8	40.5	39.9
Self-employed	52.3	38.1	50.3	51.0

SD: Standard deviation.

P values for all comparisons between intervention and control schoolchildren, by sex, were > 0.18.

### Adiposity outcomes

Changes in adiposity and cardiometabolic risk measures from baseline to the end of follow-up between intervention versus control schoolchildren, by sex, are shown in Table 2. In both sexes, prevalence of overweight or obesity fell in the IG and increased in the CG, but these changes did not reach statistical significance. Similarly, there were no statistically significant differences in a) the prevalence of underweight and the mean BMI, for both sexes, and b) in mean TST and body fat %, between the intervention and control boys. However, among girls, a reduction in indicators of adiposity in intervention versus control schools was observed, including TST (−1.1 cm; 95% CI −2.3 to −0.7; P = 0.02) and body fat % (−0.9%; 95% CI −1.3 to −0.4; P = <0.001). An increase in fat-free mass associated with the intervention was also observed in girls (0.3 kg; 95% CI 0.1 to 0.6; P = 0.04) and boys (0.5 kg; 95% CI 0.2 to 0.9; P = 0.003).

**Table 2:** **Changes in adiposity and cardiometabolic risk factors from baseline to the end of follow-up among intervention versus control schoolchildren, by sex**

	**Girls (n = 469)**					**Boys (n = 443)**				
	**Baseline** ^**a**^	**End of follow-up** ^**a**^	**Crude change**	**Adjusted difference of intervention vs control** ^**b**^ **(95% CI)**	**p**	**Baseline** ^**a**^	**End of follow-up** ^**a**^	**Crude change**	**Adjusted difference of intervention vs control** ^**b**^ **(95% CI)**	**p**
***ADIPOSITY***										
**Overweight or obesity, %**										
Control	30.0	31.7	1.7	0.8^c^ (0.3; 1.7)	0.53	36.5	38.5	2.0	0.7^c^ (0.4; 1.2)	0.16
Intervention	33.2	32.8	−0.4			40.3	38.2	−2.1		
**Underweight, %**										
Control	7.9	7.1	−0.8	1.1^c^ (0.3; 4.7)	0.88	9.5	8.3	−1.2	0.5^c^ (0.1; 2.1)	0.38
Intervention	8.7	7.4	−1.3			6.3	7.3	1		
**BMI, kg/m** ^**2**^										
Control	18.7(3.6)	19.1 (3.7)	0.4	−0.2 (−0.4; 0.1)	0.09	19.0 (3.6)	19.3 (3.7)	0.3	0.01 (−0.1; 0.1)	0.89
Intervention	18.7 (3.5)	19.0 (3.5)	0.3			19.3 (3.6)	19.4 (3.6)	0.1		
**TST, mm**										
Control	14.5 (5.4)	17.7 (7.5)	3.2	−1.1 (−2.1; −0.7)	0.02	13.4 (5.7)	16.4 (8.3)	3.0	−0.6 (−1.9; 0.8)	0.43
Intervention	15.7 (5.2)	18.2 (7.6)	2.5			14.8 (5.6)	17.3 (8.2)	2.5		
**Body fat %**										
Control	26.3 (6.0)	26.3 (6.0)	0.0	−0.9 (−1.3; −0.4)	<0.001	23.8 (8.3)	23.6 (7.0)	−0.2	−0.5 (−1.2; 0.1)	0.13
Intervention	26.6 (5.8)	25.9 (5.8)	−0.7			24.2 (6.9)	23.5 (6.7)	−0.7		
**Fat-free mass, kg**										
Control	26.7 (4.8)	28.5 (5.2)	1.7	0.3 (0.1; 0.6)	0.04	28.0 (4.7)	29.6 (5.2)	1.5	0.5 (0.2; 0.9)	0.003
Intervention	26.5 (4.6)	28.6 (4.9)	2.1			28.1 (4.6)	29.8 (4.5)	1.7		
**Waist circumference, cm**										
Control	66.2 (8.8)	68.5 (9.1)	2.3	−2.7 (−4.5; −0.9)	0.01	68.1 (9.3)	69.9 (9.9)	1.8	−1.4 (−2.6; −0.1)	0.03
Intervention	67.1 (8.9)	67.4 (8.4)	0.3			68.1 (9.7)	68.7 (9.2)	0.6		
***CARDIOMETABOLIC RISK FACTORS***										
**LDL-C, mg/dl**										
Control	94.1 (23.1)	90.7 (22.2)	−3.5	−3.8 (−6.9; −0.7)	0.015	92.9 (20.7)	90.9 (20.9)	−2.0	−2.7 (−6.9; 1.5)	0.21
Intervention	100.7 (24.2)	94.3 (23.1)	−6.4			101.1 (24.5)	97.1 (22.3)	−3.9		
**TGL/HDL-C, mg/dl**										
Control	1.3 (0.9)	1.3 (0.9)	−0.1	0.0 (−0.2; 0.2)	0.92	1.1 (0.8)	1.0 (0.7)	−0.1	0.4 (0.1; 0.8)	0.02
Intervention	1.3 (1.0)	1.3 (1.0)	0.1			1.1 (0.8)	1.2 (1.2)	0.1		
**Insulin,** **μU/mL**										
Control	8.1 (4.1)	9.7 (7.3)	1.6	−2.3 (−3.7; −0.8)	0.002	7.1 (5.5)	7.9 (6.9)	0.8	−0.04 (−2.3; 2.2)	0.97
Intervention	9.0 (5.4)	8.9 (5.0)	−0.1			7.6 (3.9)	8.0 (7.2)	0.4		
**CRP, mg/L**										
Control	1.4 (2.4)	1.6 (2.5)	0.2	0.1 (−0.5; 0.7)	0.83	1.4 (2.1)	1.7 (2.5)	0.3	−0.3 (−1.3; 0.8)	0.62
Intervention	1.5 (2.1)	1.7 (2.2)	0.2			1.4 (2.1)	1.6 (2.3)	0.2		
**MAP, mm Hg**										
Control	75.1 (6.3)	76.5 (6.9)	1.4	−0.2 (−2.5; 2.2)	0.89	75.8 (7.4)	76.8 (7.3)	1.04	0.7 (−1.8; 3.1)	0.60
Intervention	74.0 (7.6)	76.3 (6.9)	2.3			74.7 (6.9)	77.2 (6.0)	2.45		
**TG/HDL-C, mg/dl**										

BMI: Body mass index; TST: Triceps skinfold thickness; LDL-C: LDL-cholesterol; TG/HDL-C: Triglycerides / HDL-cholesterol; CRP: C-reactive protein; MAP: Mean arterial pressure.

^a^Figures at baseline and end of follow-up correspond to crude data. ^b^Differences adjusted for baseline value, age, Tanner change and cluster (random effect) using generalized mixed linear models. ^c^Odds ratio of overweight or obesity, and of underweight, among intervention versus control children.

### Cardiometabolic risk outcomes

Compared with the control group, in the intervention group a lower increase was observed in the mean WC in both boys (−2.7; 95% CI −4.5 to −0.09; P = 0.01) and girls (−1.4; 95% CI −2.6 to −0.01; P = 0.03), but only in girls did the insulin levels (−2.3; 95% CI −3.7 to −0.8; P = 0.002) and the LDL-cholesterol (−3.8; 95% CI −6.9 to −0.7; P = 0.015) decrease. The intervention was not associated with statistically significant changes in C-reactive protein or blood pressure in either sex, whereas the triglycerides/HDL-cholesterol ratio rose by 0.4 (95% CI 0.1 to 0.8; P = 0.02) in the intervention-versus-control boys, but not in the girls (Table 2).

Among fifth-grade schoolchildren, the results for the adiposity indicators and cardiometabolic risk factors did not materially change after adjustment for either caloric intake or fat intake. Lastly, interaction testing, to determine whether or not the intervention effects varied with sex, baseline BMI, Tanner stage or parental employment status, did not achieve statistical significance (data not shown).

### Process evaluation

#### Satisfaction/compliance

312 out of the 469 participating children (66.5%), and 286 (61%) of their parents completed a questionnaire to evaluate satisfaction with the program activities. In total, 99.2% of parents reported that they were fairly or very satisfied with the program, and 95.5% of the children reported that it was rarely or never necessary to remind them that they should go to the MOVI-2 program sessions. An additional file shows additional information on indicators of satisfaction and compliance with the PA program (please see Additional file 1).

#### Physical activity intensity and energy expenditure

During the intervention schoolchildren performed an average of 49.0 min/day of moderate-vigorous intensity PA, while control children did an average of 46.5 min/day. Sedentary time was higher in intervention children (956.5 min/day) than in their control counterparts (876.7 min/day).

The average energy expenditure during each session, estimated by indirect calorimetry, was 4.2 kcal/minute. The mean energy expenditure per week, resulting from the PA included in MOVI-2, was estimated as 1357 kcal (SD = 447). The children’s average heart rate in each session was 151 beats · min^-1^.

#### Sustainability

The cost of our intervention was 28 euros/month per children, similar to English reinforcement or music classes. As the program cost was wholly subsidised by the research grant, participation in the program was free of charge.

#### Adverse outcomes

Dizziness during baseline venipuncture occurred in 2% of the children at baseline, and in 1.1% of the children at the end of the study. No other adverse events were reported by students during health examinations. Two minor ankle sprains occurred during the sessions of the program (9 months incidence risk: 0.4 %). One boy was expelled from the program for aggressive behavior toward peers; his parents and the School Board made the decision by consensus.

## Discussion

### Main findings

After controlling for baseline variables, we did not observe a significant effect of the intervention on the prevalence of overweight/obesity. However, the intervention was associated with significant reductions in various indexes of adiposity in girls, and with a significant increase of fat-free mass in both genders. Furthermore, children of the intervention group improved their cardiometabolic risk profile although the effects were more visible in girls. Specifically, the intervention was associated with a significant decrease: a) in TST, body fat %, WC, insulin and LDL-cholesterol levels in girls. However only WC was reduced in boys while their TG/HLD-c ratio actually increased with the intervention. No important adverse events were registered, and the cost of the program was comparable to that of other common extracurricular activities of children in Spain (e.g., English language or music). Finally, children’s and parents’ satisfaction levels with the program were high.

### Comparison with other studies

#### Adiposity outcomes

The intervention programs aimed to reduce adiposity in children are often use as outcome variables changes in BMI and other measures of adiposity. It is known that changes in BMI may reflect changes on lean body mass rather than on fatness, especially when young people are engaged in PA interventions. Thus it is not surprising that effective programs report reductions in adiposity measured by skinfolds thickness or body fat %, yet fail in reducing BMI. Our intervention was associated with an increase of fat free mass in both genders, but also with reductions in body fat % and in TST that were significant only in girls, this changes in girls may have been influenced by sexual maturity, but the interaction for Tanner stage in our analyses did not achieve statistical significance. A similar previous intervention tested in the same schools also showed similar results [20], even though intensity and weekly duration were greater in the current study. However, a three years exercise intervention in Copenhagen schools that consisted of doubling the time of physical education classes [33] failed to produce statistically significant changes in body composition parameters. Differences in duration, frequency and intensity of the intervention, and its mandatory nature, could be behind the differences in adiposity outcomes between studies.

#### Cardiometabolic risk outcomes

Observational studies reporting the relationship between PA and cardiometabolic risk in children are inconclusive [34]. Most intervention studies focus on reducing MetS risk or insulin resistance in obese children and/or adolescents. The improvements on adiposity or cardiorespiratory fitness found in these studies were accompanied by decline in adiposity indicators. In contrast, school-based intervention studies are scarce, and their effectiveness is greater in children with excess of body fat [16]. Gender subgroup analysis in most of these intervention studies is lacking. In a previous intervention study testing the effectiveness of the MOVI program our group found that this PA program was successful in improving lipid profile, especially in girls [20,35]. The current study extend our previous findings in that we currently report that MOVI-2 is also effective in reducing cardiometabolic risk profile in girls, as a result of a marked decline in WC, insulin levels and LDL-cholesterol. According to results of other studies [14], greater emphasis on muscular strength exercises in MOVI-2 sessions could be responsible for this success in improving insulin resistance levels. It is becoming evident that muscle-strengthening activities are major determinants of muscular strength [36]. In turnmuscular strength has been described as a positive predictor of insulin sensitivity in children [37], probably as a result of changes in muscle quality (strength/unit of muscle mass; increased areas of type I and type II fibers) [38].

Three systematic reviews agree that the effectiveness of PA interventions on improving lipid profile is far from being clarified [12,34,39]. Contrary to what we expected in light of data from a similar intervention [35], the lipid profile in intervention boys worsened while improved in CG. Itt is difficult to ascertain the circumstances behind this finding, particularly when the WC increase was greater in the CG. We can only speculate about the reasons for this finding. Perhaps,resistance exercise leading to reduced waist circumference could increase lipid mobilization of abdominal adipose tissue, thereby increasing blood triglycerides [40]. Moreover, considering both that the effect of physical activity on lipids are acute, lasting only 1–3 days [41], and that our program finished on May, 30th, and other municipality’s programs involving mostly boys from de CG (i.e. soccer school) usually last until latter, it might be that the effect of our program in IG had been diluted, and no the effect of programs involving boys from CG [33]. Lastly, probably in relation to the difficulties in accurately measuring blood pressure in field trials of healthy children, the effectiveness of PA interventions on reduction of blood pressure in normotensive children remains controversial [39,42]. As in previous interventions, the MOVI-2 program has failed to reduce blood pressure levels in schoolchildren, and particularly MAP.

Several reasons can be argued for explaining why the effectiveness of the MOVI-2 program in decreasing adiposity and cardiometabolic risk is greater in girls than in boys, including that the sport activities promoted by local councils in rural areas were mainly soccer and basketball, for which boys have greater predilection than do girls. Moreover, it has recently been suggested that PA interventions have had only a small effect on children’s overall PA levels [43], which explains why such interventions have had limited effectiveness on reducing adiposity. This argument may be relevant to our data, since in our program, as in others [17], boys may have compensated for their increased PA in the MOVI-2 by reducing their sports activity outside the program. However other interventions [33] have improved cardiometabolic risk in boys but not in girls; the only, but substantial, difference between both studies is that our intervention was more effective on reducing waist circumference and other adiposity parameters in girls than in boys.

Nevertheless, the gender differences in effectiveness, as observed in our school-based PA intervention and others [20,33,35], raise a serious dilemma as to which values should prevail when designing PA interventions in the school environment: educational values based on gender equity, or functionalist values based on efficiency in the management of resources; in other words, which criteria should prevail, be they ethical or educational criteria, or cost effectiveness criteria. Moreover, it is difficult to guess what would have happened if the PA intensity of the program’s sessions had been higher, because the dropout rate would probably also had been higher, and the program might not be effective in boys and lose the effectiveness in girls.

#### Compliance and satisfaction with the program

Data from a previous recreational non-competitive intervention showed a very low rate of dropouts and a high level of satisfaction of the children with the program [20]. In MOVI −2 program compliance rates were acceptable but not as higher as in the previous program, probably because the Saturday sessions were irreconcilable with family activities on the weekend; however both parents and children showed high levels of satisfaction in both versions of the MOVI program, probably due to the playful nature of both interventions.

Although the MOVI-2 program included PA of more vigorous intensity and longer duration than MOVI, the current intervention not produced greater health benefits. It is due, probably, to the low adherence of sessions of Saturday morning mentioned above.

### Strengths and limitations of the study

Most of the interventions and systematic reviews aimed to reduce adiposity or cardiometabolic risk in children have not reported gender differences with regard to effectiveness [11,16,17,44,45]. However, it has been suggested that girls and boys do not necessarily respond comparably to a given intervention [46]. Our study reveals that the success of school-based PA interventions could be very different between girls and boys, probably because baseline PA levels are greater in boys, and therefore the relative increase of the same intervention is smaller for this gender group. Moreover, until now, the effectiveness of PA interventions in schoolchildren has been reported in urban settings, but studies in rural areas are scarce, probably assuming that sedentary behaviours in children living in those areas are less frequent, bit this statement is far from the reality [47].

Most interventions to prevent obesity in children lack examination of how they influence underweight prevalence. Our intervention, given that it consisted of promoting PA but was not aimed to reduce caloric intake, did not significantly change the underweight prevalence, a problem whose magnitude is increasing in Spain [2]. Our intervention has estimated the average of energy expenditure—in METS—which is attributable not only to the sessions as a whole but also to each game included in them (please see http://www3.uclm.es/proyectosCESS/movi2/public/energy). Finally, some interventions that addressed specific subgroups of population, such as immigrants, girls or the obese, are, in our opinion, susceptible to stigmatization of the target group. In comparison, our intervention may be highly generalizable given that it takes place in the school environment, does not exclude any children by gender, ethnic group or physical condition (except in cases of severe diseases or mental disorders), and its cost (28 euros/month per children) is similar to other free-time activities in Spain, such as English, music lessons or attendance to other PA programs as dance or athletics schools. It was not possible the comparison with other public health initiatives aimed at reducing cardiometabolic risk in children because they are of nutritional nature and their cost have been not reported [29].

Our study has several limitations. First, it was conducted mainly in rural schools, thus findings still need to be confirmed in urban settings. Second, although the follow-up period was not too much longer, extended over an additional academic year, the duration of the intervention presumably would not change its success substantially, as has been proven in a previous similar intervention [35]. Third, anthropometric and blood pressure determinations were not blinded to intervention allocation. It should be noted, however, that our study included only highly reproducible primary end points; furthermore, weight, body fat %, and blood pressure were measured using automatic digital devices, which reduced observer error. Fourth, it has been advocated that interventions in children are more effective when mandatory [48], but our intervention could be considered obligatory for the children who agreed to participate, as it was scheduled as part of the afternoon activities offered by the school. Finally, we have only estimates of daily PA in a subsample of 200 participants that wore an accelerometer for 7 days; in these estimates not statistically significant gender differences in moderate-vigorous PA were found, but the measurement of other compensatory sedentary behaviors might have been necessary.

## Conclusions

Overall, in light of our data, the MOVI-2 program has shown that a 1-year extracurricular intervention with non-competitive PA aimed at all fourth- and fifth-grade schoolchildren is safe and effective in reducing adiposity in both genders, and in improving insulin cardiometabolic risk in girls.

Finally, most of the PA interventions aimed to improve cardiometabolic risk in children and adolescents are actually a ‘black box’, which limits seriously its generalisability. The characteristics of our intervention include, among others, being open, generalizable, sustainable and acceptable for parents and children.

This work has shown that a 1-year extracurricular intervention with non-competitive PA aimed at all fourth- and fifth-grade schoolchildren, regardless of body weight, is safe and effective in reducing adiposity. Future research should assess whether similar interventions are effective in younger and older children. Renewed efforts should also be devoted to devising interventions with the potential to reduce cardiometabolic risk factors in children.

## Additional file

Additional file 1:
**Process indicators of the MOVI-2 physical activity program.**
