# Supplementary material for: Gender differences on effectiveness of a school-based physical activity intervention for reducing cardiometabolic risk: a cluster randomized trial
Source: Int J Behav Nutr Phys Act. 2014 Dec 10;11:154. doi: 10.1186/s12966-014-0154-4 (PMC4295398; doi:10.1186/s12966-014-0154-4)
Supplement: Additional file 1: — Process indicators of the MOVI-2 physical activity program. [file 12966_2014_154_MOESM1_ESM.doc]

**Additional file 1**. Process indicators of the MOVI-2 physical activity program.

| **Attendance/dropout**  66.7% of schoolchildren attended more than 70% of the program sessions  49 (10.4%) children withdrew from the program because of the following reasons:   - Problem behaviors (aggression to peers or monitor) - Changes of residence - Incompatibility with other activities - As punishment by parents for poor academic performance of children. |
| --- |
| **Oversight measures**   - Two meetings were conducted with monitors; one at baseline and the other one at 3 months after the beginning of the program. - Monthly contacts with monitors were held by phone and e-mail to obtained information on attendance of schoolchildren to the program. - 3 visits (one per quarter) to the centers were made to assess program performance and conduct satisfaction surveys among children. |
| **Satisfaction/compliance**  312 (66.5%) children completed questionnaires to evaluate satisfaction with the program activities:   - 96.1% reported that they always or almost always liked the games played in MOVI-2. - 95.5% reported that they rarely or never needed to be reminded to go to the MOVI-2 session. - 94.8% reported that they always or almost always felt happy with the monitor. - 92.2% reported that they always or almost always felt loved by the group.   286 (61%) parents completed questionnaires to evaluate satisfaction with the program activities:   - 99.7% reported that the monitor always or almost always arrived on time. - 100% indicated that the monitor always or almost always kept control of the group. - 99.7% reported that the monitor promoted a good climate in the classroom. - 98.2% reported that their children were always or almost always interested in attending MOVI-2. - 97.2% indicated that they were always or almost always happy with the monitor. - 90.9% reported that the program was one of their children's most important activities. - 99.2% responded that they were satisfied or very satisfied with the program. |
